# A combination of modified atmosphere packaging and two chemical disinfectants: Effects on microbial, sensory, and physicochemical properties of raw ready‐to‐eat leek

**DOI:** 10.1002/fsn3.3047

**Published:** 2022-09-15

**Authors:** Behnam Hosseininezhad, Marzieh Nader, Asghar Ramezanian, Mehrdad Niakousari, Seyed Mohammad Mazloomi

**Affiliations:** ^1^ Department of Food Hygiene and Quality Control School of Nutrition and Food Sciences, Shiraz University of Medical Sciences Shiraz Iran; ^2^ Department of Horticultural Science School of Agriculture, Shiraz University Shiraz Iran; ^3^ Department of Food Science and Technology School of Agriculture, Shiraz University Shiraz Iran; ^4^ Food and Supplements Safety Research Center Shiraz University of Medical Sciences Shiraz Iran

**Keywords:** food safety, postharvest, quality evaluation, ready to eat, vegetable

## Abstract

In the present study, the effect of modified atmosphere packaging (MAP) on microbial (total aerobic count, yeasts and molds, Enterobacteriaceae, psychrotrophic bacteria, and lactic acid bacteria), physicochemical (pH, moisture content, and color), and sensory properties of raw ready‐to‐eat leek was investigated after disinfection using Percidin–Nanosil disinfectants. There were six different treatments, of which four samples were packaged as MAP (T_1_ to T_4_) and two samples were packaged as non‐MAP (T_5_ and T_6_). The highest and the lowest reduction in the number of microorganisms after disinfection was observed in psychrotrophic bacteria and total aerobic count, respectively. The count of microbial variables in T_2_ sample (87% N_2_, 3% CO_2_, and 10% O_2_) was lower than in other treatments. No significant difference was observed in the physcochemical properties (moisture and pH) of treated samples except for the color of T_6_ sample (cling film) compared to other treatments (*p* < .05). It is noteworthy that the panelists did not recognize differences among T_1_, T_2_, and T_5_ samples. However, T_2_ treatment was effective in maintaining microbial, physicochemical, and sensory properties of leek compared to the other treatments. Also, T_6_ sample showed the lowest quality in all parameters. Disinfection combined with MAP was able to maintain the physicochemical, microbial, and sensory properties of raw ready‐to‐eat leek during storage.

## Graphical abstract

The disinfection alongside modified atmosphere packaging (hurdle effect) was able to maintain physicochemical, microbial and sensory properties of raw ready‐to‐eat leek (in T2 sample) during storage.

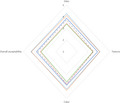

## 1 INTRODUCTION

Fruits and vegetables constitute an important part of the human diet (Yousuf et al., 2018) and are good sources of biologically active compounds, which may prevent some chronic diseases (Granado‐Lorencio et al., 2008). Leek (*Allium porrum* L.) is one of the most commercial leafy vegetables in the world. It belongs to the *Allium* genus (*Alliaceae* family) along with onion and garlic (Ozgur et al., 2011). The shelf life of fresh ready‐to‐eat vegetables is greatly limited by the increase in metabolic activity, color destruction, and microbial growth (Char et al., 2012). A wide range of microorganisms including bacteria, yeasts, and molds affect the safety and quality of fresh vegetables and fresh‐cut fruits. Gram‐negative bacteria especially *Pseudomonas* and *Enterobacteriaceae* species constitute 80 to 90% of bacterial flora. Lactic acid bacteria (LAB) can be the natural intrinsic flora of fruits and vegetables which can be correlated with fresh product spoilage and unpleasant odors. The number of yeasts and molds (YM) is usually smaller than bacteria; however, as they grow in large numbers, they can contribute to soft rot spoilage and decomposed fermented products (Oliveira et al., 2015).

Washing with water can lower the potential of contamination, although it can transfer pathogenic microorganisms (Gil et al., 2009). Disinfecting agents are known to ensure quality and safety in the food industry (Tirpanalan et al., 2011). Their efficiency varies depending on the type, dosage, and duration of the disinfecting process (Yarahmadi et al., 2012). Peracetic acid (acetic acid–hydrogen peroxide) solution with the chemical formula of C_2_H_4_O_3_ is introduced into the market with several brand names such as Proxitane, Prasan, and Percidin. This achromatic liquid, which belongs to the broad‐spectrum oxidizing compounds family, can eradicate bacteria, viruses, fungi, and spores and is considered a cold sterilizer (Najafi & Arbabi, 2013). Another disinfectant is commercialized with the commercial name of Nanosil (hydrogen peroxide–silver ion) (Barikgugjlu et al., 2016). Nanosil is composed of hydrogen peroxide similar to Percidin. As an advantage, it does not have any dangerous side effects on both humans and the environment (Khazaei et al., 2008).

To package ready‐to‐eat food products, modified atmosphere packaging (MAP) can be employed. The concept of this method is to package a perishable product in an atmosphere containing a different mixture of atmosphere gases (Mousavi‐Baigi & Sedaghat, 2014). Oxygen (O_2_), carbon dioxide (CO_2_), and nitrogen (N_2_) are the most commonly used gases in MAP (Oliveira et al., 2015). Nitrogen has three functions in MAP: displacement of O_2_ to delay oxidation, postponing of the growth of aerobic spoilage organisms, and acting as a filler to maintain package conformity (Farber et al., 2003). The effectiveness of CO_2_ as an antimicrobial agent depends significantly on the temperature, type of microorganism, growth phase, water activity, and chemical composition of the product (Putnik et al., 2017). The use of MAP for fresh‐cut products requires careful selection of the film and package type for each commodity (Oliveira et al., 2015). Important factors in the selection of suitable packaging film include clarity, mechanical strength, machinability, seal ability, printability, and gas barrier properties (Farber et al., 2003). Nowadays, in the development of newly packaged ready‐to‐eat products with extended shelf‐life, it is recommended to use multiple methods simultaneously because of the “hurdle effect.” This study aimed to evaluate the shelf life, physicochemical and sensory characteristics, and microbiological safety of fresh leek disinfected by commercial Percidin (peracetic acid) and Nanosil, packaged as MAP.

## 2 MATERIALS AND METHODS

### 2.1 Leek preparation and disinfection

Fresh leeks were prepared from a local farm located in Shiraz, Iran (during the months of October and November 2019), and processed the same day under aseptic conditions. Following the removal of physically damaged, dehydrated, or discolored (yellowed) leaves, leek stalks were cut. At the beginning of the experiment, 10 g of raw leeks were subjected to microbial evaluation. The leek stalks were immersed in the distilled water for 5 min, afterward treated with 0.66 ml/L Percidin (acetic acid–hydrogen peroxide) for 3 min (according to manufacturer's instructions) and 3.75 ml/L Nanosil (hydrogen peroxide ‐silver ion) for 30 minutes (according to manufacturer's instructions). The disinfected samples were drained in a laminar flow biosafety cabinet until no visible water droplets were present and then a 10 g sample was taken for microbial evaluation.

### 2.2 Modified atmosphere packaging

Washed and disinfected fresh leeks were placed in 60 g portions in polypropylene (PP) trays (15 cm width × 20 cm length × 3 cm depth). Two commercial moisture absorbent pads (60 ml/L) were placed in each tray in order to absorb any condensed moisture and to keep samples fresh. Modified atmosphere packaging (DZ‐400, Wenzhou Zhonghuan Packaging Machine Co., Ltd, China) of these trays was then performed using a two‐layer laminated film (20 cm width × 30 cm length; 90 μm thickness) made of polyester (PS) as the outer layer and polyethylene (PE) as the inner layer. The permeability of the film to oxygen was 113 ml.μm/m^2^.day.Kpa.

A total of six different treatments (Table 1) were applied in two replicates. T_1_ to T_4_ treatments included MAP by injecting a mixture of gases (oxygen, carbon dioxide, and nitrogen). Two other treatments (T_5_ and T_6_) were packaged using ambient air. T_5_ group was sealed in the PS/PE plastic bag as the “MAP control” sample and T_6_ sample was packaged using cling film as the package control sample. The packages were stored at 5.0 ± 0.5°C for 9 days. All microbial experiments, including total aerobic count, yeasts and molds, *Enterobacteriaceae*, psychrotrophic bacteria, and lactic acid bacteria, as well as sensory evaluation, were carried out on days 2, 6, and 9 of the experiment. Physicochemical characteristics including pH, moisture content, and color were measured on days 1, 5, and 9 of the experiment.

**TABLE 1:** Packaging treatments used in this study.

Sample	N_2_%	CO_2_%	O_2_%	Packaging material
T_1_ (MAP)	67.00 ± 0.10	3.00 ± 0.10	30.00 ± 0.10	PS/PE
T_2_ (MAP)	87.00 ± 0.10	3.00 ± 0.10	10.00 ± 0.10	PS/PE
T_3_ (MAP)	94.00 ± 0.10	3.00 ± 0.10	3.00 ± 0.10	PS/PE
T_4_ (MAP)	30.00 ± 0.10	0.00 + 0.01	70.00 ± 0.10	PS/PE
T_5_ (Control –Ambient air)	78.00 ± 0.01	0.04 ± 0.01	21.00 ± 0.01	PS/PE
T_6_ (Cling film –Ambient air)	78.00 ± 0.01	0.04 ± 0.01	21.00 ± 0.01	Cling film

### 2.3 Microbial evaluation

Microbiological determinations were done using 10 g sample of leek per replicate on days 2, 6, and 9. The samples were mixed with 90 ml of sterile normal saline in a sterile bag and homogenized in a laboratory stomacher (Easy Mix, AES Chemunex) for 90 s. Regarding psychrotrophic bacteria, cultured plate count agar (PCA) Petri dishes were incubated for 7 days at 7.0 ± 0.5°C, and for the total aerobic count test, they were incubated for 3 days at 30.0 ± 0.5°C. *Enterobacteriaceae* were determined using violet red bile glucose agar (VRBG) incubated at 37 ± 0.5°C for 1 day. Lactic acid bacteria (LAB) were enumerated on De Man–Rogosa–Sharpe Agar (MRS), followed by incubation at 37 ± 0.5°C for 48 h. Yeast and mold count (incubated for 5 days at 25 ± 0.5°C) were determined on yeast extract glucose chloramphenicol agar (YGC).

### 2.4 Physicochemical evaluation

#### 2.4.1 pH

Leek samples (10 g) were homogenized in distilled water (100 ml/L) using a blender (LB20ES, Waring) and the pH of the solution was measured using a pH meter (Metrohm 827, Switzerland). Three measurements were taken on every sample. The results were expressed as units of pH (Feldsine et al., 2002).

#### 2.4.2 Moisture content

Moisture content was determined gravimetrically according to the 984.25 AOAC method (Feldsine et al., 2002) in duplicate, and results were expressed as a percent. Around 3 g of a homogenized sample was dried at 103 ± 2°C (Oven OT 140, Iran) to constant weight.

(1) $$\left({W^{*}}_{1}-{W^{*}}_{2}/{W^{*}}_{1}\right)\times 100$$

Where W*_1_ is the sample weight before heating and W*_2_ is the sample weight after heating.

##### Color measurement

The color of leek samples was determined using a Konica Minolta Colorimeter (CR 400, Minolta). The results were expressed in terms of L*, a*, and b* Hunter Lab values, where L* represents the lightness, a* represents chromaticity on a green (−) to red (+) axis, and b* represents chromaticity on a blue (−) to yellow (+) axis. Also, the total color difference (∆E) between color parameters of the samples on day 1 and day 9, for each sample, was calculated using the following equation:

(2) $$∆E=\sqrt{{\left(L_{1}^{*}-L_{9}^{*}\right)}^{2}+{\left(a_{1}^{*}-a_{9}^{*}\right)}^{2}+{\left(b_{1}^{*}-b_{9}^{*}\right)}^{2}}$$

Where *L* ^*^_1_, *a* ^*^_1_, and *b* ^*^_1_ are the color parameters of the samples on day 1 and *L* ^*^_9_, *a* ^*^_9_, and *b* ^*^_9_ are the color parameters of the samples on day 9.

### 2.5 Sensory evaluation

Nine untrained panel members aged 22–40 years were recruited among the students, faculty, and staff of the School of Nutrition and Food Sciences, Shiraz, Iran. The samples were evaluated under white illumination at room temperature. Leek characteristics including texture, aroma, color, and overall acceptability were scored on a 1–5 scale, where 5 = very good, 4 = good, 3 = normal, 2 = poor quality, and 1 = not acceptable (Waghmare & Annapure, 2013).

### 2.6 Statistical analysis

Statistical analyses were performed using SPSS 26 (IBM SPSS Software) and Microsoft Excel. All microbial counts were log‐transformed for statistical analyses. The microbial evaluation was performed via Freidman nonparametric test, and for comparing the days for each treatment, Kruskal–Wallis test (*p* < .01) was run. The Mann–Whitney U nonparametric test was employed to analyze the significance of the difference (disinfecting effect) for all features. Physicochemical properties (pH, moisture, and color) of samples were tested by repeated measures and Duncan's comparison test (*p* < .05). In addition, the analysis of sensory test results was performed using the Kruskal–Wallis test.

## 3 RESULTS

### 3.1 Microbiology enumeration

#### 3.1.1 Evaluation of the efficacy of disinfectants

Raw leeks were treated with 0.66 ml/L Percidin (acetic acid–hydrogen peroxide) for 3 minutes and 3.75 ml/L Nanosil (hydrogen peroxide–silver ion) for 30 minutes before packaging. Microbial load in intended treatment (B) reduced compared to raw leek (A). Total aerobic count, yeasts and molds, *Enterobacteriaceae*, psychrotrophic bacteria, and LAB were decreased by 0.37, 0.48, 0.69, 1.05, and 0.77 log cfu g^−1^, respectively. The most and least amount of microbial load reduction was reported for the psychrotrophic bacteria and *Enterobacteriaceae* (Table 2).

**TABLE 2:** Efficacy of disinfectants on the microbial load of raw leek.

Factor	A	B	*p*‐value
	Mean ± SD (log CFU g‐^1^)	Mean ± SD (log CFU g‐^1^)	
Total aerobic count	6.59 ± 0.308	5.814 ± 0.368	<0.01
Enterobacteriaceae	4.739 ± 0.510	4.362 ± 0.380	0.008
Lactic acid bacteria	4.089 ± 0.389	3.606 ± 0.544	0.002
Yeasts and molds	4.640 ± 0.254	3.950 ± 0.579	<0.01
Psychrotrophic bacteria	5.978 ± 0.326	4.921 ± 0.434	<0.01

*Note*: A: Before washing + disinfection; B: After washing + disinfection.

#### 3.1.2 Effects of MAP on microorganisms

**FIGURE 1:**
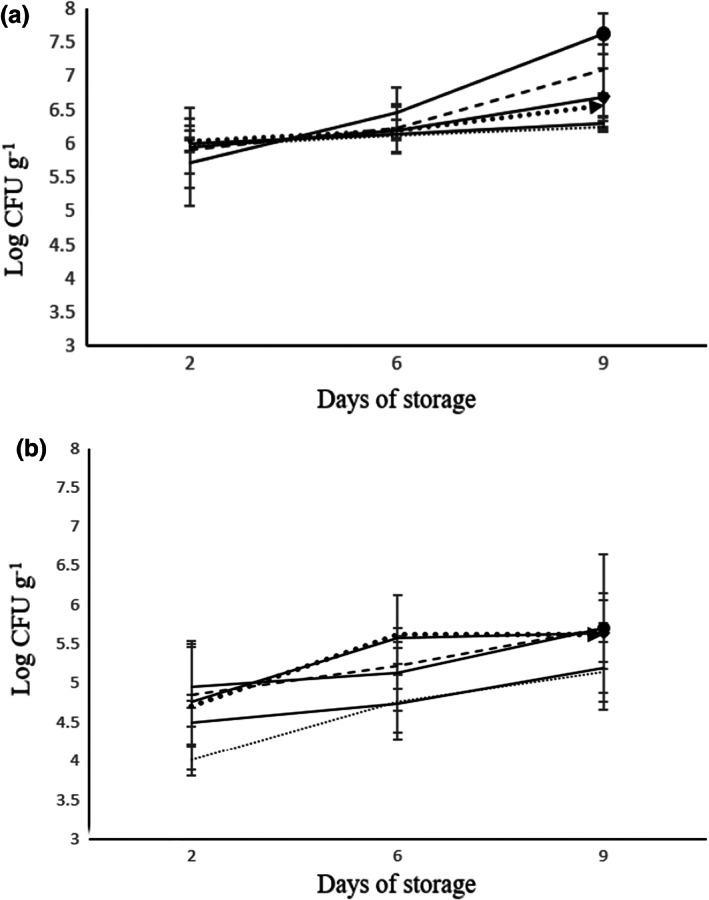
The effect of modified atmospheric packing on the total number of aerobic bacteria (a), yeasts, and molds (b) during 9 days of storage at 5°C. T_1_: (67% N_2_, 3% CO_2_, 30% O_2_) (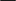), T_2_: (87% N_2_, 3% CO_2_, 10% O_2_) (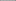), T_3_: (94% N_2_, 3% CO_2_, 3% O_2_) (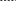), T_4_: Super atmospheric oxygen (SAO) (30% N_2_, 0% CO_2_, 70% O_2_) (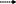), T_5_: Control sample (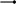), and T_6_: Cling film (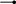).

**FIGURE 2:**
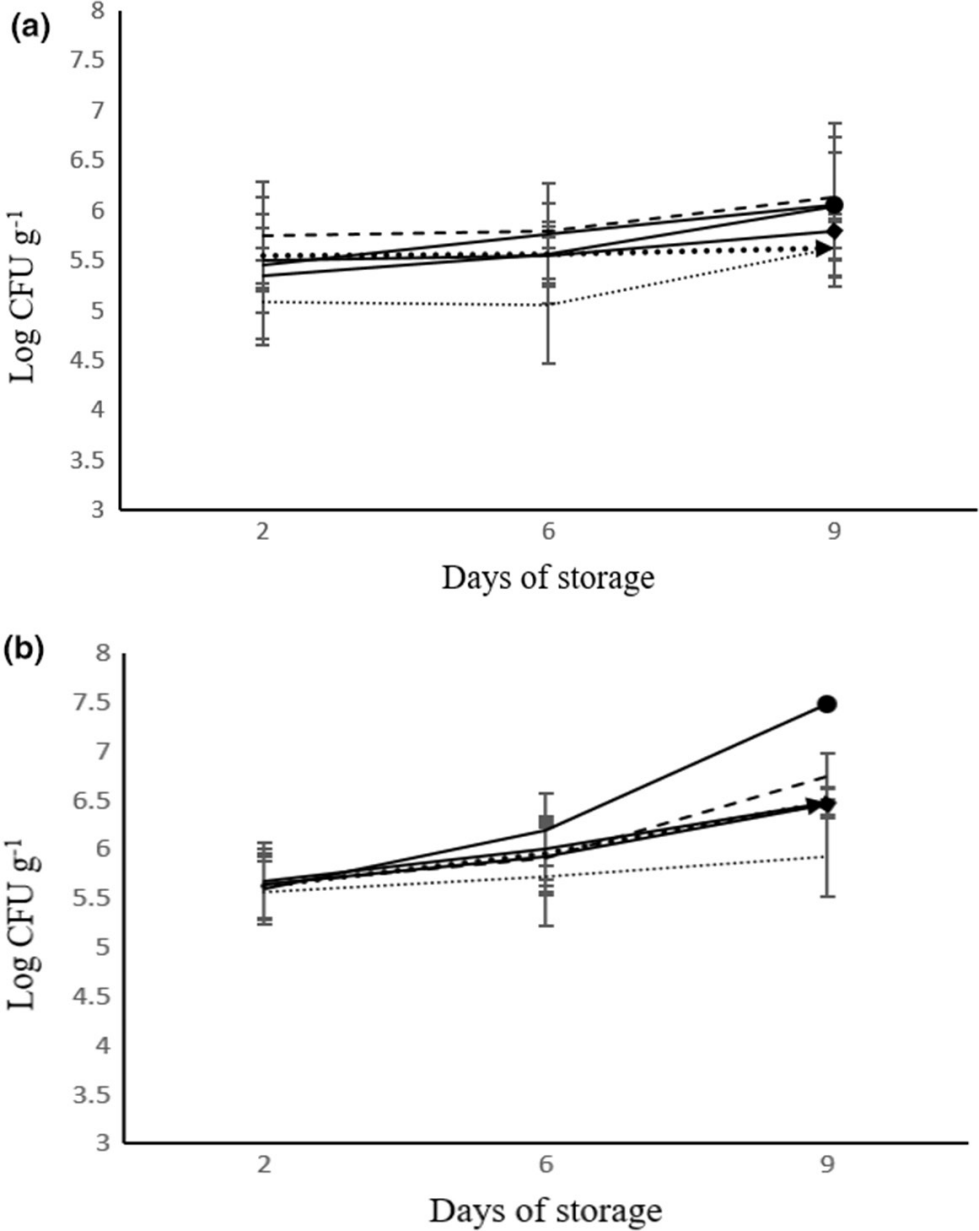
The effect of modified atmosphere packaging on *Enterobacteriaceae* (a) psychrotrophic bacteria (b) during 9 days of storage at 5°C. T_1_: (67% N_2_, 3% CO_2_, 30% O_2_) ( 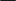 ), T_2_: (87% N_2_, 3% CO_2_, 10% O_2_) (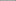), T_3_: (94% N_2_, 3% CO_2_, 3% O_2_) (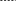), T_4_: Super atmospheric oxygen (SAO) (30% N_2_, 0% CO_2_, 70% O_2_) (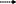), T_5_: Control sample ( 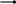 ), and T_6_: Cling film ( 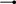 ).

During the storage period, increases in the microbial count were observed in all samples. At the end of the storage, the number of yeasts and molds for different treatments was different and there was a significant difference between them (*p* < .01). The highest and lowest contents of yeasts and molds were related to T_6_ (5.7 log cfu g^−1^) and T_2_ (5.1 log cfu g^−1^) samples, respectively (Figure 1b). The number of LAB in the leek's samples was increased in all treatments after the storage time ranged between 4.09 and 4.79 log cfu g^−1^. The lowest and highest count of LAB were observed in T_2_ (4.09 log cfu g^−1^) and T_4_ samples, respectively (4.79 log cfu g^−1^) (Figure 2).

**FIGURE 3:**
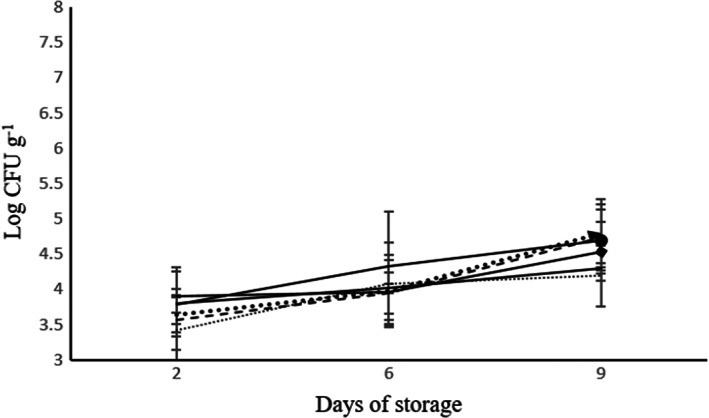
The effect of modified atmosphere packaging on lactic acid bacteria during 9 days of storage at 5°C. T_1_: (67% N_2_, 3% CO_2_, 30% O_2_) ( 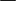 ), T_2_: (87% N_2_, 3% CO_2_, 10% O_2_) (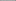), T_3_: (94% N_2_, 3% CO_2_, 3% O_2_) (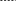), T_4_: Super atmospheric oxygen (SAO) (30% N_2_, 0% CO_2_, 70% O_2_) (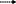), T_5_: Control sample ( 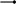 ), and T_6_: Cling film ( 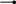 ).

After 9 days of storage at 5°C, *Enterobacteriaceae* count in different samples was 5.6–6 log cfu g^−1^ with the highest count in T_6_. The microbial count of *Enterobacteriaceae* was controlled in T_2_ (87% N_2_, 3% CO_2_, and 10% O_2_) and T_4_ samples (30% N_2_, 0% CO_2_, and 70% O_2_) which contained superatmospheric oxygen (SAO). Initially, the count of psychrotrophic bacteria ranged from 5.5–5.6 log cfu g^−1^, and after the end of storage time, it reached 5.9–7.4 log cfu g^−1^. T_6_ sample showed the highest amount of psychrotrophic bacteria. T_2_ sample controlled the psychrotrophic bacteria better than other treatments; it showed a nonsignificant difference on different days (Figure 3b). After the storage period, the total aerobic count in T_1_ to T_6_ samples was 6.3, 6.2, 7.1, 6.5, 6.6, and 7.6 log cfu g^−1^, respectively. The total aerobic count of all treatments increased and there was a significant difference in microbial load in different treatments after the end of shelf life (*p* < .01) (Figure 1a).

### 3.2 pH and moisture content

**FIGURE 4:**
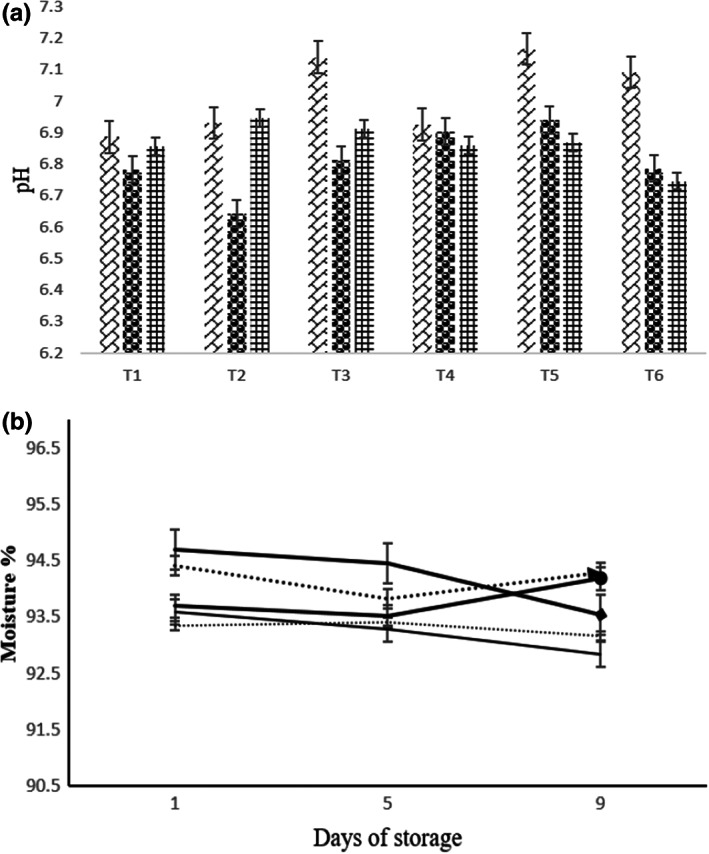
(a) The pH of the samples was measured using pH meters. Day 1: ( 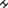 ), day 5: ( 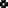 ), and day 9: ( 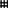 ). (b) Moisture (%) according to AOAC 984.25 method in the samples kept at 5 °C. T_1_: (67% N_2_, 3% CO_2_, 30% O_2_), T_2_: (87% N_2_, 3% CO_2_, 10% O_2_), T_3_: (94% N_2_, 3% CO_2_, 3% O_2_), T_4_: (30% N_2_, 0% CO_2_, 70% O_2_), and T_5_: control sample, T_6_: cling film (*p* < .05).

The pH of all samples except T_2_ sample with a slight increase declined during storage at 5°C. The pH of the samples was between 6.7 and 6.9. There was no significant difference among days and treatments at the end of the storage period. (Figure 4a).

The moisture content of samples had a significant difference on different days, but the interaction between the treatment and the storage time showed no significant difference (*p* < .05). The moisture content of samples was between 92.84 and 94.19% at the end of the storage period. (Figure 4b).

### 3.3 Color changes

**FIGURE 5:**
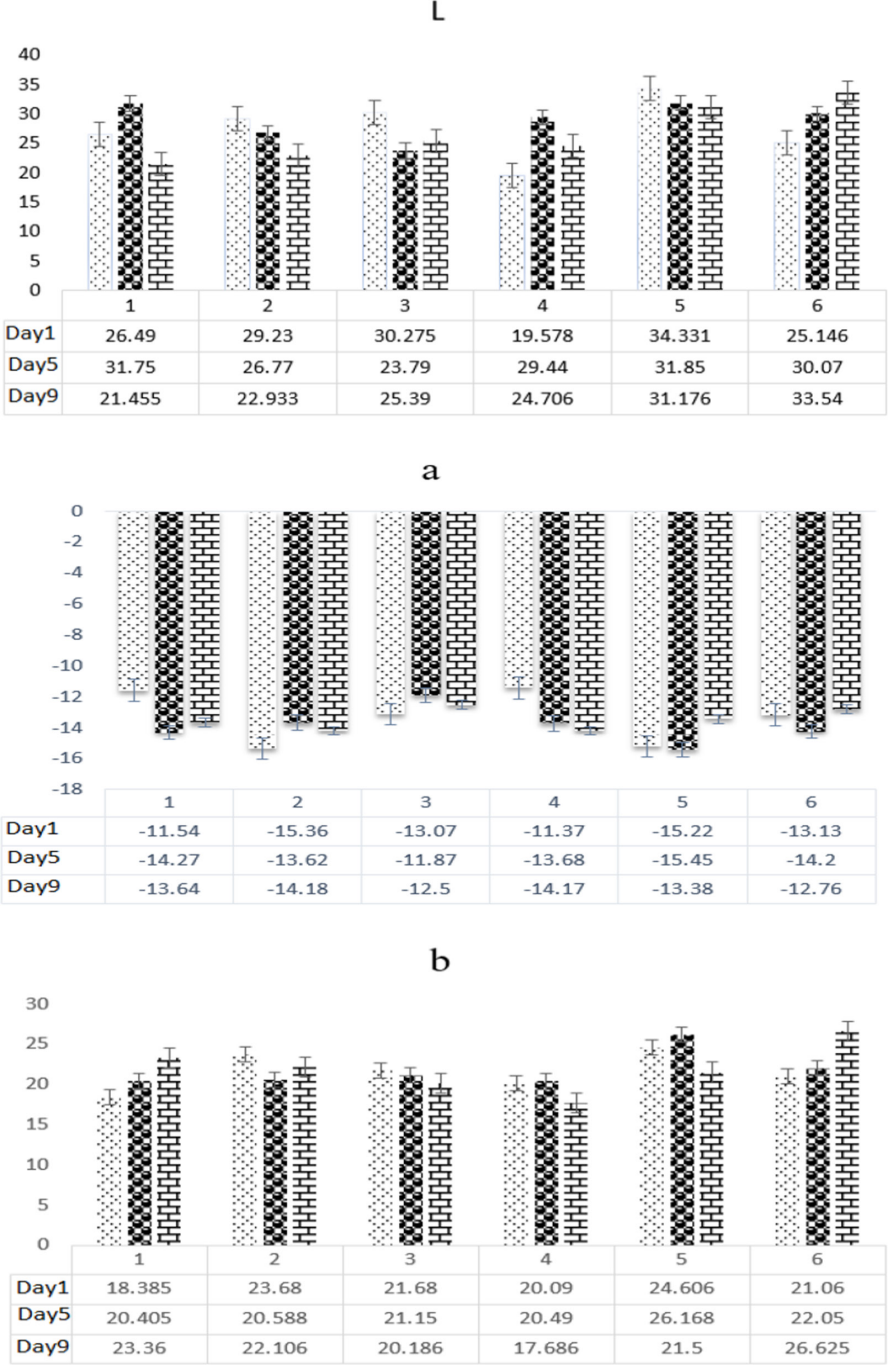
The L, a, and b values of leek samples on days 1, 5, and 9 of storage at 5°C. Day 1: ( 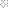 ), Day 5: ( 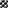 ), and Day 9: ( 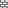 ).

Statistical analysis showed significant differences (*p* < .05) for L* value, among the treatments, interaction of treatments, and storage time. The lightness (L*) in the T_6_ sample was raised to the highest level, which showed the positive effect of (MAP) packaging in maintaining this color parameter (Figure 5). The a* value also showed a decreasing trend during the storage. A significant reduction in the b* value was also observed during storage. The total color difference (∆E) of the samples was measured with T_5_ (control) and T_6_ (cling film) samples as a reference after 9 days of storage.

Based on the data, among the MAP samples (T_1_ to T_4_), T_2_ and T_3_ samples had a narrower color difference than the others, after 5 and 9 days, respectively. T_6_ samples showed the most color change after 9 days of storage. Nonetheless, the T_5_ sample showed the lowest ∆E. A comparison of the color difference between T_5_ and other MAP samples (∆ET_5_) also showed that T_3_ sample had a minor color difference from the control sample after 9 days of storage. This means the modified atmosphere had a positive effect on this parameter compared to cling film packaging (Table 3).

**TABLE 3:** Total color difference (∆E) between the fresh samples (day 1) and the samples after 5 (∆E_D5_) and 9 (∆E_D9_) days of storage; and total color difference of the samples after 9 days of storage with T_5_ (∆E_T5_) and T6 (∆E_T6_) samples as reference.

Sample	∆E_D5_	∆E_D9_	∆E_T5_	∆E_T6_
T_1_	6.266092	7.402701	9.902399	12.54764
T_2_	4.326678	6.604704	8.304573	11.61587
T_3_	6.61689	5.141433	5.998241	10.38957
T_4_	10.13688	6.320539	7.55229	12.64573
T_5_	2.933993	4.796814	0	5.677076
T_6_	5.135274	10.07281	5.677076	0

*Note*: T_1_: (67% N_2_, 3% CO_2_, 30% O_2_), T_2_: (87% N_2_, 3% CO_2_, 10% O_2_), T_3_: (94% N_2_, 3% CO_2_, 3% O_2_), T_4_: (30% N_2_, 0% CO_2_, 70% O_2_), T_5_: control sample, T_6_: cling film.

### 3.4 Sensory evaluation

**FIGURE 6:**
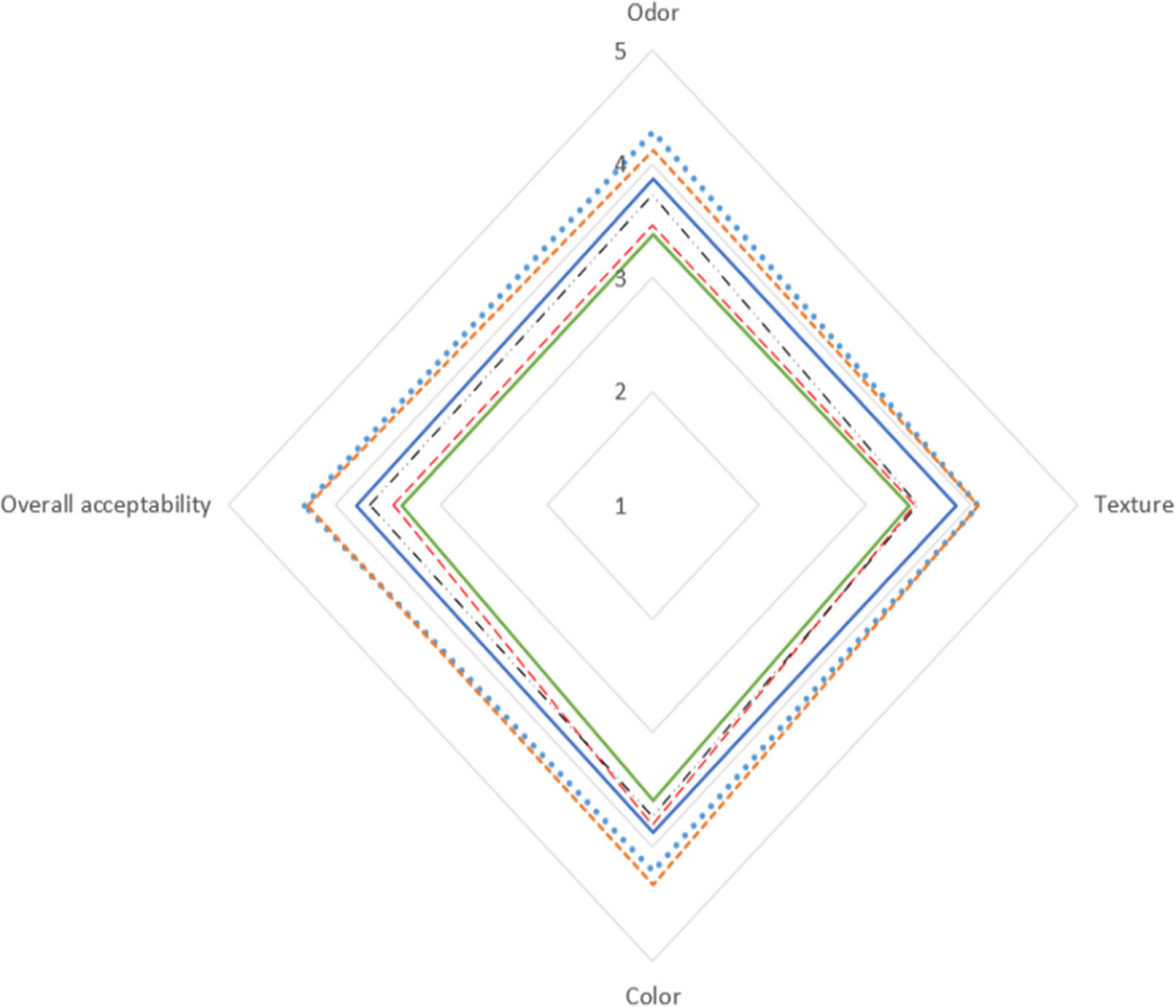
The effect of modified atmosphere packaging on sensory attributes of leek during storage at 5 °C for 9 days. T_1_: (67% N_2_, 3% CO_2_, 30% O_2_), T_2_: (87% N_2_, 3% CO_2_, 10% O_2_), T_3_: (94% N_2_, 3% CO_2_, 3% O_2_), T_4_: (30% N_2_, 0% CO_2_, 70% O_2_), T_5_: control sample, and T_6_: cling film (*p* < .05). T_1_: ( 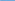 ), T_2_: ( 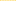 ), T_3_: ( 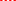 ), T_4_: (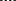), T_5_: ( 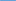 ), T_6_: ( 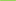 ).

Figure 6 represents the odor, texture, color, and overall acceptability scores for all the treated and nontreated leek. The most important parameter in the sensory evaluation of leek was the smell and tissue of leek. All the treatments showed quality reduction during the storage period. Measurement of sensory parameters was an indicator of significant difference among treatments (*p* < .05). The highest overall acceptability score was related to T_1_, T_2_, and T_5_, and the lowest was related to T_6_ sample.

## 4 DISCUSSION

This study was conducted to improve the quality of leek using a combination of disinfectant (Percidin–Nanosil) and MAP during storage. Microorganisms are one of the most important factors contributing to the reduction in shelf life and the quality of vegetables. Therefore, it is necessary to suppress the growth of microorganisms (Murcia et al., 2009). A wide range of disinfectants are currently used to reduce the microbial load of fresh produce, and each of them has its own advantages and disadvantages. The efficiency of disinfectants depends on various factors, such as dosage, contact time, and type of product. In the present study, Percidin and Nanosil disinfectants led to a significant reduction of the total aerobic count, and psychrotrophic bacteria, yeasts, and molds. Leeks were first disinfected by Percidin due to its unpleasant odor (Barikgugjlu et al., 2016).

In a study conducted by Vandekinderen et al. (2009), disinfection of freshly chopped leek using 80 ml/L of disinfectant for 60 s reduced the initial microbial contamination of leeks (between 5.58 and 7.29 log cfu g^−1^) by 1 log cfu g^−1^. Barikgugjlu et al. (2016) investigated the effect of Nanosil (120, 150, and 180 ppm) and peracetic acid (40, 60, and 80 ppm) on the level of *Salmonella* enteritidis and *E. coli* O157: H7 on raw mixed vegetables (coriander, parsley, basil, coriandrum, spearmint, leek, and radish). The contact times (5 and 10 min) showed no significant difference in the reduction in test organisms. At the highest concentration, peracetic acid reduced the number of *Salmonella enteritidis* and *E. coli* O157: H7 by 1.65 and 1 log cfu g^−1^, respectively. The reduction values by Nanosil were 2.24 and 2.04 log cfu g^−1^, respectively. Overall, Nanosil showed higher activity against test organisms. In another study, the total microbial count of freshly cut strawberries reduced by 2.6 log cfu g^−1^ after disinfection with 100 ml/L of peracetic acid for 120 s (Van de Velde et al., 2016). Dai et al. found a reduction of 2.16 and 2.20 log cfu g^−1^ in the total aerobic count after disinfection of freshly chopped scallion with peracetic acid at the concentrations of 150 ml/L (2 min) and 100 ml/L (5 min), respectively (Dai et al., 2012).

MAP approach is applied to reduce respiration of fresh produce without leading to anaerobic respiration; as a result, the oxygen content is generally recommended to be between 1 and 5%. The low oxygen content in the packages may lead to the production of ethanol, acetaldehyde, and organic acids which is accompanied by discoloration, texture change, and unpleasant taste (Granado‐Lorencio et al., 2008).

The count of LAB was at the highest level in T_3_ sample (with very low oxygen content) compared to the other treatments because they were microaerophilic bacteria. The total aerobic count was at the highest level in packaging groups with low CO_2_ content (in treatments of T_3_ to T_5_, and T_6_). It seems that the antimicrobial properties of CO_2_ can be an effective factor in reducing the total aerobic count. Among all treatments, T_2_ group (87% N_2_, 3% CO_2_, and 10% O_2_) showed lower microbial content after 9 days of storage. As expected, the growth of yeasts and molds (aerobic microorganisms) was at the highest level in T_4_ sample (with the highest oxygen level). However, different treatments showed no significant differences in pH and moisture content.

In terms of color, peracetic acid as an oxidizing agent had no negative effect on leek (T_1_ to T_5_ groups). Therefore, T_6_ group exhibited a significant difference from the other groups, suggesting a positive effect of packaging film and MAP. Tsouvaltzis et al. (2008) reported that the amount of L* value (lightness parameter) in minimally processed leek in control packaging (ambient air) decreased further compared to the MAP packaging (1% O_2_ + 14% CO_2_) during 14 days of storage at 6.5°C.

Sensory evaluation done by the untrained panelists showed better overall acceptability of all samples of leek till day 6, but on day 9, it was difficult to distinguish any difference among T_1_, T_2_, and T_5_ samples. In the study of Gómez and Artés (2005), the sensory evaluation of celery sticks in two MAP packaging conditions (6 kPa O_2_ + 7 kPa CO_2_ and 9 kPa O_2_ + 5 kPa CO_2_) was better maintained than the control (ambient air) sample at 5°C. There are several studies on the efficacy of MAP approach alone or in combination with chemicals in shelf‐life extension. Exama et al. (1993) reported that O_2_ concentrations of less than 1% and CO_2_ concentrations of more than 15% are critical concentrations for agricultural products. However, the critical concentrations for each product depend on several variables such as temperature, physiological conditions, maturity, and preparation processes.

In a study, the effect of MAP approach on the freshly cut leek was investigated. The results showed that storing leek at 5°C under 5% O_2_, 5% CO_2_, and 90% N_2_ had moderate effectiveness (Mousavi‐Baigi & Sedaghat, 2014). Arvanitoyannis et al. (2011) investigated microbiological and sensory properties of rocket salad (Arugula) alone and in combination with “Lollo Verde” lettuce under MAP conditions (5% O_2_ and 10% CO_2_ as MAP‐A treatments and 2% O_2_ and 5% CO_2_ as MAP‐B treatments) for 10 days. The color was better preserved in both MAP treatments compared with the passive sample.

Rosa et al. (2007) studied the effect of the improved atmosphere (88% N_2_, 2% CO_2_, and 10% O_2_) and inactive atmosphere packaging on the physicochemical, microbial, and sensory properties of the freshly cut parsley during storage at 5 °C. They showed that parsley had better quality stability under inactive atmosphere packaging compared with MAP conditions during a 6‐day time period. The inefficiency of MAP in increasing parsley shelf life can be at least partly attributed to different respiration rates of products which can even be affected by season.

In the previous research, MAP approach (5% O_2_, 10% CO_2_, and 85% N_2_) showed higher efficacy in improving the physicochemical and sensory properties of freshly cut papaya during 25 days of storage at 5 °C when used in combination with chemicals emphasizing the use of “hurdle technology” in food preservation (Feldsine et al., 2002). Barbosa et al. (2016) investigated the effect of three MAP treatments with very high CO_2_ and very low or zero O_2_ contents on the physicochemical and sensory properties of the cooked vegetables. They found a nonsignificant difference in pH, moisture, and color in different treatments and they observed no significant difference between the samples in terms of sensory parameters.

## 5 CONCLUSIONS

In this study, the effect of Percidin–Nanosil disinfectants was evaluated along with MAP during 9 days of storage at 5 °C. The results showed that among different treatments, T_2_ sample (87% N_2_, 3% CO_2_, and 10% O_2_) had the best microbial, physicochemical, and sensory properties. Also, the lowest quality among the treatments in all variables was related to T_6_ sample. The shelf life of leek without using disinfectants and MAP was limited to 6 days. Therefore, the disinfection alongside MAP (hurdle effect) was able to maintain physicochemical, microbial, and sensory properties of raw ready‐to‐eat leek during storage.

## CONFLICT OF INTEREST

The authors declare that there is no conflict of interest regarding the publication of this study.

## Data Availability

The data that support the findings of this study are available from the corresponding author upon reasonable request.
